# Changing Pattern of Organ Donation and Utilization in the USA

**Published:** 2012-11-01

**Authors:** R. F. Saidi

**Affiliations:** *Division of Organ Transplantation, Department of Surgery, University of Massachusetts Medical School, Worcester, MA, USA*

**Keywords:** Organ transplantation, End-stage organ failure, Brain-dead donor, Living donors

## Abstract

**Background: **Organ transplantation has proven highly effective in the treatment of various forms of end-stage organ failure. However, organ shortage is still the greatest challenge facing the field of organ transplantation.

**Objective:** To assess the pattern of organ donation and utilization during the past decade in the USA.

**Methods:** We studied OPTN/UNOS database for organ donation between January 2000 and December 2009. The retrieved records were then categorized into two time periods—from January 2000 to December 2004 (era 1), and from January 2005 to December 2009 (era 2).

**Results:** There were 65,802 living and 71,401 deceased donors in the US from 2000 to 2009, including 66,518 (93.2%) brain-dead donors and 4,883 (6.8%) donation after cardiac death. Comparing two periods—from January 2000 to December 2004 (era 1) and from January 2005 to December 2009 (era 2), the number of deceased donors increased by 25% from 31,692 to 39,709 and living donors decreased by 7.6%. Donation after cardiac death increased from 3.5% to 9.3%. The portion of donors older than 64 years increased from 6.9% in era 1 to 11.3% in era 2 (p=0.03). The number of donors with a body mass index of >35 kg/m^2^ was also increased from 6.8% to 11.2%. A significant increase in the incidence of cardiovascular/cerebrovascular as cause of death was also noted from 38.1% in era 1 to 56.1% in era 2 (p<0.001), as was a corresponding decrease in the incidence of death due to head trauma (34.9% *vs*. 48.8%). The overall discard rate also increased by 41% from 13,411 in era 1 to 19,516 in era 2. This increase in discards was especially more prominent in donation after cardiac death group which rose by 374% from 440 in era 1 to 2,089 in era 2. The discard rate for livers and kidneys increased by 31% and 68%, respectively, comparing era 1 and era 2. We noted a 78% increase for discarded donation after cardiac death livers and 1,210% for discarded donation after cardiac death kidneys.

**Conclusion:** We detected significant changes in the make-up of the donor pool over the past decade in the US. Over time, donor characteristics have changed with increased numbers of elderly donors and donors with comorbidities, especially donors who died of cardiovascular/cerebrovascular disease. The incidence of donation after cardiac death has increased significantly; brain-dead donors have only increased slightly and living donors have decreased. As the result, the discard rates have increased. The transplant community and policy makers should consider every precaution to safeguard the donor pool and prevent the decay of organ quality in favor of quantity.

## INTRODUCTION

The greatest challenge facing the field of organ transplantation today is to increase the number of organs available for transplant. A variety of approaches have been implemented to expand organ donor pool including increased live donation, a national effort to expand deceased donor donation, split organ donation, paired donor exchange, national sharing models and greater utilization of expanded criteria donors. Although donation after brain death (DBD) accounts for the majority of deceased organ donors, in the recent years, there has been a growing interest in donors who have severe and irreversible brain injuries but do not meet the criteria for brain death. If the physician and family agree that the patient has no chance of recovery to a meaningful life, life support can be discontinued and the patient can be allowed to progress to circulatory arrest and then still donate organs (donation after cardiac death [DCD]).

In the last 10 years, the number of deceased organ donors has increased nationally by 40%, whereas DCD has increased 10-fold with almost 800 cases of DCD reported in 2008 [1-3]. In this study, we examined the pattern of donation and utilization in the US over the past ten years (2000 to 2009) by reviewing the Organ Procurement and Transplantation Network/United Network for Organ Sharing (OPTN/UNOS) database.

## MATERIALS AND METHODS

We performed a retrospective analysis of OPTN/UNOS database about who were consented for organ donation between January 2000 and December 2009. The retrieved records were then categorized into two time periods—from January 2000 to December 2004 (era 1), and from January 2005 to December 2009 (era 2). The results were based on data reported to OPTN/UNOS as of October 25, 2010. Discard was considered when the organ was recovered but not transplanted. Statistical comparisons were made using χ^2 ^test. A p value <0.05 was considered statistically significant.

## RESULTS

**Figure 1:**
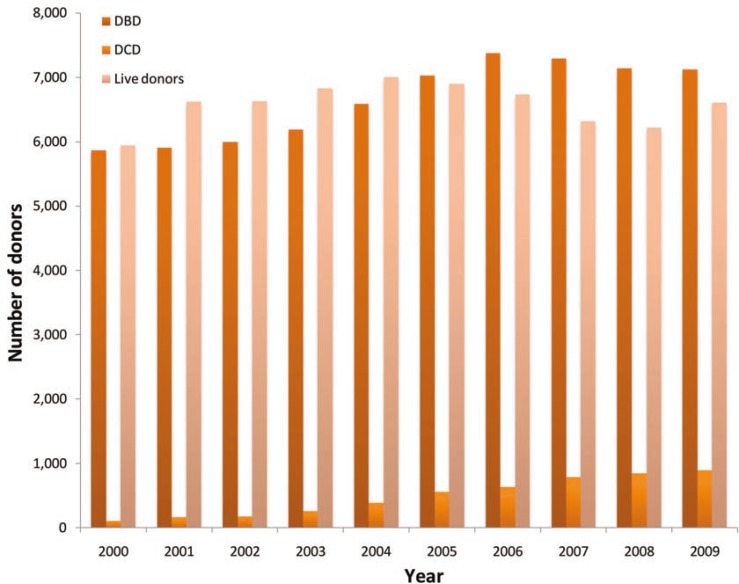
Number of donors in the US (2000–2009)

There were 68,802 living donors and 71,401 deceased donors in the US from 2000 to 2009, including 66,518 (93.2%) DBD and 4,883 (6.8%) DCD. The number of DCD increased from 117 (1.9%) in 2000 to 901 (10.9%) in 2009 (Fig. 1). Comparing the two studied periods (Table 1), era 1 (01/2000–12/2004) and era 2 (01/2005–12/2009), the number of deceased donors increased by 25% from 31,692 to 39,709; DCD increased from 3.5% to 9.3%; the number of DBD rose by 17% comparing era 1 (n=30,557) to era 2 (n=35,961). At the same time, the number of DCD rose by 230% from 1135 in era 1 to 3748 in era 2. The DBD had a peak in 2006 and constantly decreased after that from 7375 in 2006 to 7294 in 2007, 7142 in 2008, and to 7121 in 2009 (Fig. 1). On the other hand, the number of living donors decreased by 7.6% from 33,026 in era 1 to 32,776 in era 2. The decrease in the number of living donors was more prominent since 2004 which peaked at 7004 (Fig. l); it has not reached that level since.

**Table 1:** Comparing donors characteristics in era 1 (2000–2004) and era 2 (2005–2009)

	**Era 1**	**Era 2**	**p**
Live donors	33026	32776	0.06
Deceased donors	31692	39709	0.02
DBD	30557	35961	0.2
DCD	1135 (3.5%)	3748 (9.3%)	<0.01
Donor age >65 yrs	6.9	11.3%	0.03
Donor BMI >35 kg/m^2^	6.8%	11.2%	0.03
COD			<0.01
CVA/CVD	38.1%	56.1%	
COD Trauma	48.8%	34.9%	
Anoxia	12.6%	8.4%	
CNS tumor	0.5%	0.6%	

COD: Cause of death

CVA: Cerebrovascular disease

CV: Cardiovascular disease

**Figure 2:**
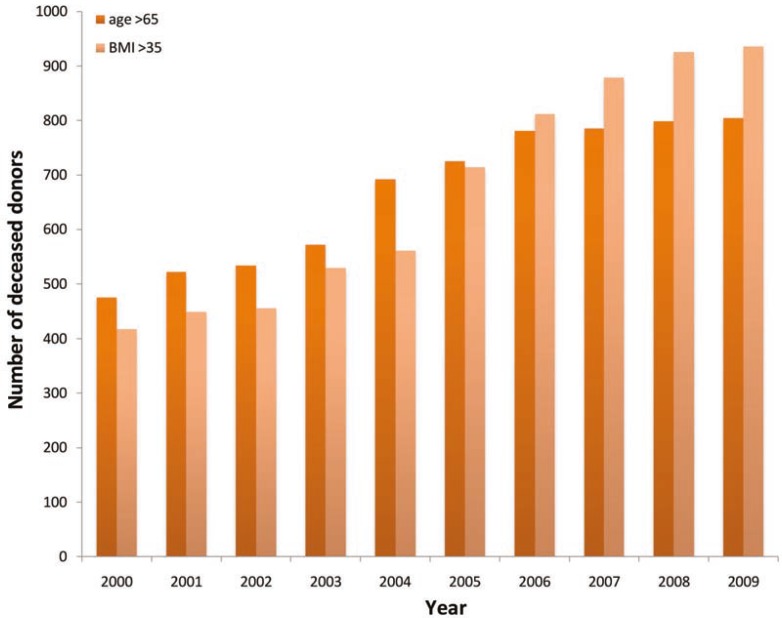
Number of deceased donors older than 65 years or with a BMI >35 kg/m^2^ (2000–2009)

There has also been a change in the demographics of organ donors over the course of this study. As shown in Table 1, a significant increase in the incidence of cardiovascular/cerebrovascular, as cause of death, was increased from 38.1% in era 1 to 56.1% in era 2 (p<0.001); there was a concomitant decrease in the incidence of death due to head trauma (34.9% *vs*. 48.8%). The proportion of donors older than 64 years almost doubled from 6.9% in era 1 to 11.3% in era 2 (p=0.03). The number of donors with a body mass index (BMI) >35 kg/m^2 ^also increased from 6.8% to 11.2% (Fig. 2).

**Figure 3:**
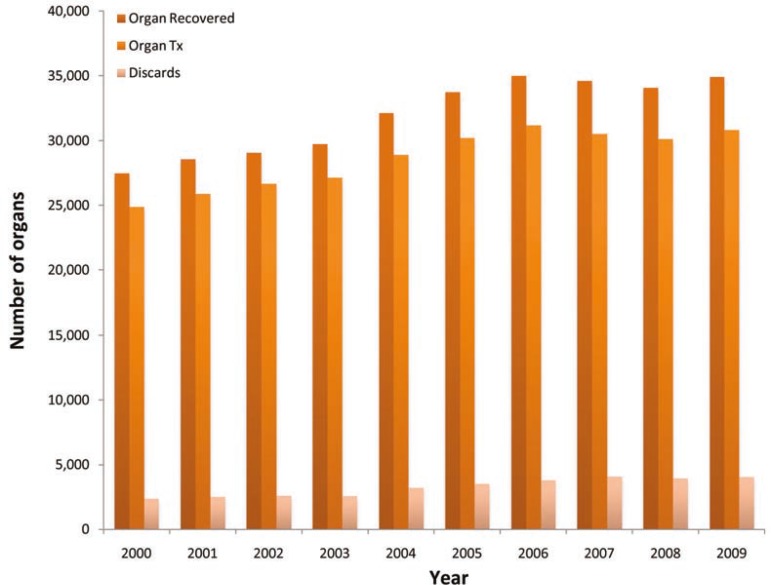
Organs recovered, transplanted and discards in the US (2000–2009)

Figure 3 shows the pattern of the overall number of organs recovered and transplanted from 2000 to 2009. The total number of organs recovered and transplanted increased from era 1 to era 2. There were 146,923 organs recovered in era 1 which increased by 41% to a total of 207,366 in era 2. On the other hand, the total number of organs transplanted increased by 37% from 133,508 in era 1 to 183,991 in era 2. However, the overall discard rate increased by 41% from 13,164 in era 1 to 19,516 in era 2 (**Table 2**). This increase in discards was especially more prominent in DCD group which rose by 374% from 440 in era 1 to 2089 in era 2. The discard rate for livers, and kidneys increased by 31%, and 68%, respectively, especially in the DCD group. We noted 78% increase in the discarded DCD livers and 1210% in DCD kidneys. The number of organs recovered decreased for 4.5 organs per donor in 2000 to 4.3 organ per donor in 2009. On the other hand, the number of organs transplanted per donor decreased from 4.1 in 2000 to 3.8 in 2009. As the result, the number of organs recovered and transplanted per donor decreased form 4.5 and 4.1 in era 1 to 4.3 and 3.8 in era 2, respectively.

**Figure 4:**
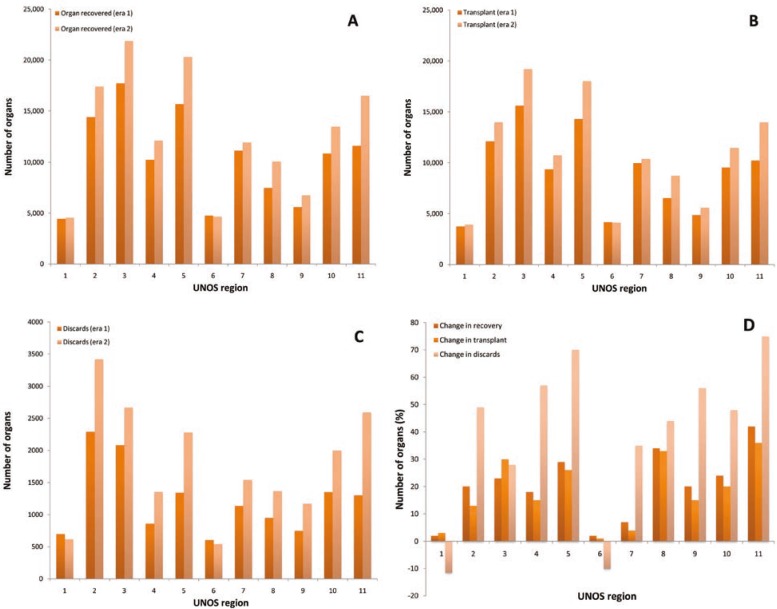
Organ recovery (A), transplanted (B), discards (C) and changes in organ recovered, transplanted and discarded (D), comparing different UNOS regions in era 1 and era 2.

We also observed a very similar pattern of organ recovery, transplanted and discarded in 11 UNOS regions, comparing era 1 with era 2 (Fig. 4). The number of organs recovered, and transplanted increased in all regions (Fig. 4A and 4B) from 2%–42%, and 1%–36%, respectively. On the other hand, the discards increased by 20%–70% in different region, except for regions 1 and 6 where discards decreased by 12%, and 10%, respectively (Fig. 4C and 4D).

## DISCUSSION

Organ transplantation has proven highly effective in the treatment of various forms of end-stage organ failure. Increased public awareness, improved efficiency of the donation process, greater expectations for transplantation, expansion of the living donor pool and the development of standardized donor management protocols have led to unprecedented rates of organ procurement and transplantation. In 2008, more than 28,000 patients received organ transplants from more than 14,000 deceased and live donors in the US [1, 4-10]. Despite the work of the Organ Donation and Transplant Collaborative and the marked increase in the number of deceased donors early in the effort, the number of deceased donors rose by a total of only 67 from 2006 to 2007 [1].

In addition to recent stagnant growth in overall donors, the percentage of standard criteria donor (SCD) steadily declined from 78% in 1998 to about 65% in 2007 [1]. This decline can be attributed to increases in the number and percentage of ECDs and DCDs [1]. The shift in the distribution of recovered kidneys from SCD to ECD and DCD impacts utilization, since DCD and ECD kidneys have higher rates of discard. The observed increase in DCD also explains, in part, the fewer organs per donor that are recovered and transplanted overall, and the current state of less than 3.75 organs transplanted per donor (OTPD); the OTPD was 2.08 for DCD, 1.72 for ECD and 3.63 for SCD in 2007 [11-13]. In this study, we found that the number of organs recovered and transplanted per donor decreased form 4.5 and 4.1 in era 1 to 4.3 and 3.8 in era 2, respectively.

Compared to the year 2006, in 2007, 299 fewer SCD kidneys were transplanted; there was an increase of 163 DCD non-ECD transplants [1-5]. Consistent with the goals set by HRSA for DCD development, the percentage of donors from DCD continues to increase. There has been a total increase in the percentage of donors that are categorized as DCD, from 8% in 2006 to 9.8% in 2007; the number and percentages of DCD liver and kidney transplants continue to increase substantially [1-12]. We encountered parallel changes in our study; the number of DCD donors increased markedly from 3.5% in era 1 to 9.3% in era 2. On the other hand, we noted a decrease in living donation and only a slight increase in the number of DBD.

Our analysis showed a significant change in the diagnosis leading to donation over the last 10 years. There was a shift from trauma donors to donors with cardiovascular/cerebrovascular disease in the US. The portion of donors who died of trauma decreased from 48.8% in era 1 to 34.9% in era 2 (Table 1). On the other hand, donors who died of cardiovascular/cerebrovascular disease rose from 38.1% in era 1 to 56.1% in era 2. The UNOS data from 1995 shows that 48.8% of donors died of trauma and that this number decreased to 34.9% in 2008 [5].

Although the total number of donors increased by 25% from era 1 to era 2, the number of DBD peaked in 2006 and constantly decreased since (Fig. 1). The main increase in the number of donors was in DCD group which raised 230%, comparing era 1 to era 2. At the same time, the number of DBD increased by only 17%, comparing era 1 and era 2. Whether this represents addition of donors who would not have ever progressed to brain death or an exchange for DCD in cases that would have previously followed a DBD pathway, remains uncertain. If the latter is the case, this may reflect a change in clinical practice in which withdrawal of support is offered earlier in the patient’s course—before brain death occurs. Saidi, *et al.* [13], identified a significant change in resuscitative practices over time, with a striking rise in new surgical interventions such as craniostomy, craniotomy, cooling, *etc.*, that have the potential to intercede in the progression to brain death. These interventions were strongly associated with intent to donate via DCD. The lesser likelihood of making the diagnosis of brain death in these patients provides a plausible explanation for at least part of the stagnant growth of DBD compared with DCD in our program and in the region. 

The portion of donors older than 64 years increased from 6.9% in era 1 to 11.3% in era 2 (p=0.03). The number of donors with BMI >35 kg/m^2^ was also increased from 6.8% to 11.2%. The number of organs recovered and transplanted per donor decreased form 4.5 and 4.1 in era 1, to 4.3 and 3.8 in era 2, respectively. Inspection of UNOS data revealed that nationally in 2009, an average of 3.6 organs were recovered from DBD donors compared to 2.5 organs from DCD. In addition, 3.1 organs were transplanted form DBD donors compared to 1.9 from DCD. On average per 100 donors, DCD donates 20 less kidney (170 *vs*. 190), 40 less liver (40 *vs*. 80), and five less pancreas (2 *vs*. 7) than DBD [6].

As the result of increase in DCD, more donors with comorbities and elderly donors, we also noted a dramatic increase in the discard rates; the overall discard rate increased by 41% from 13,411 in era 1 to 19,516 in era 2. This increase in discards was especially prominent in DCD group which rose by 374% from 440 (20.9%) in era 1 to 2089 (24.9%) in era 2. The discard rate for livers and kidneys increased by 31% and 68%, respectively, especially in DCD group—78% for DCD livers and 1210% for DCD kidneys.

The data on DCD organ recovery rates were compounded by the large and ever-growing literature indicating that organs recovered from DCD donors may have a compromised outcome post-transplant [14, 15]. Allograft and patient survival of DCD kidneys are reported to be similar to DBD kidneys; however, DCD kidneys have been associated with increased resource utilization [16]. For DCD livers, there is a high rate of biliary strictures that have been attributed to the period of warm ischemia that occurs between withdrawal of donor life support and organ preservation. This leads to a reduction in graft survival and an increase in the need for re-transplantation [15]. For heart, lung, and pancreas recipients there is little utilization of DCD organs, though some centers have reported acceptable outcomes using DCD pancreata [17-19].

The transplant community should come up with solutions to deal with problems such as optimal identification and management of DCD donors and more investment in live donation. There should also be emphasis on measures to improve the quality of organs like pumping the organs or resuscitation of DCD organs [20, 21]. Organ allocation and distribution has its roots in the heterogeneous and somewhat arbitrary geographic boundaries that determine the current donation service areas (DSA) and UNOS regions. This has led some to call for broader allocation units to make distribution more equitable and not only based so tightly on the geography. This can potentially lead to better utilization of organs and also decrease the discard rate [22]. Our study also showed that there was a wide variation in different regions regarding changes in or organ recovery (2%–42%), transplantation (1%–36%) and discards (10% to 70%). The transplant community and policy makers should consider every precaution to safeguard the donor pool and prevent the decay of organ quality in favor of quantity.
